# Characterization of different solid fuels from waste for an advanced online fuel control system designed for large-scale incineration plants

**DOI:** 10.1177/0734242X231178224

**Published:** 2023-06-14

**Authors:** Jürgen Oischinger, Markus Kohl, Martin Meiller, Julian Walberer, Robert Daschner, Andreas Hornung, Florian Grafmans, Ragnar Warnecke, Robert Breitenberger, Franz Dannerbeck, Martin Zwiellehner

**Affiliations:** 1 Fraunhofer UMSICHT, Fraunhofer Institute for Environmental, Safety, and Energy Technology, Sulzbach-Rosenberg, Germany; 2 Department of Chemical and Biological Engineering, Faculty of Engineering, Friedrich-Alexander University Erlangen-Nuremberg, Erlangen, Germany; 3 Gemeinschaftskraftwerk Schweinfurt GmbH (GKS), Schweinfurt, Germany; 4 SAR GmbH, Prozess- und Umwelttechnik, Dingolfing, Germany

**Keywords:** fuels, laser, volume measurement, bulk density, lower heating value (LHV), advanced online fuel control system, waste incineration, municipal solid waste incineration (MSWI), large-scale incineration plants

## Abstract

Despite many years of experience in the incineration of solid fuels from waste, the heterogeneity of solid fuels and their varying properties still pose a challenge for a stable and clean combustion in large-scale incineration plants. In modern facilities such as municipal waste incineration plants there still exists a lack of knowledge on the exact amount and calorific value of waste entering onto the grate. Based on the works of Warnecke et al. and Zwiellehner et al., in our project ‘AdOnFuelControl’, we determined the initial bulk density at the feed hopper by measuring the weight of the waste via the crane weigher and the volume via a high-performance 3D laser scanner. With the help of the determined bulk density, the lower heating value (LHV) and the compression in the feed hopper were calculated. All this information was integrated into the combustion control system, which provided a high potential for an optimized operation of the plant. In this article, six different fuels (fresh and aged municipal solid waste, refuse-derived fuel (fluff), refuse-derived fuel (fine grain), waste wood and dried, grained sewage sludge) were examined for the elemental composition, the LHV, fuel-specific parameters and the compression behaviour. In addition, initial tests with the 3D laser scanner as well as formulas for the calculation of the density in the feed hopper were presented. Based on the results of the experiments, the chosen approach seems very promising for optimized combustion control in large-scale incineration plants. As a next step, the gained knowledge and technology should be integrated in the municipal waste incineration plant.

## Introduction

The combustion of heterogeneous solid fuels such as municipal solid waste (MSW), refuse-derived fuels (RDF), waste wood or other biomass on grate firings is quite challenging. Due to the heterogeneity of the fuels and their varying properties such as water content, heating value and ash content, an unstable incineration process can occur resulting in fluctuating thermal output, thermal overload in the combustion chamber or exceedance of emission values. In modern facilities, the firing control regulates the combustion process by changing the length of the stroke, frequency of the ram feeder and the grate bars and the amount, and the distribution and the temperature of the primary and secondary combustion air. Nevertheless, the fuel mass flow onto the grate and the heating value of the fuel cannot be measured directly. Normally, the fuel mass flow is calculated by a several-hour averaged value of the crane weigher. Hence, the combustion control always operates with values from the past or responds to drastic changes in single parameters in the system (Aleßio, 2012; Horeni et al., 2007; Zwiellehner et al., 2018a, 2018b). Consequently, a predictive combustion control, which could measure online the amount and quality of the caloric value (CV) of the fuel passing on the grate, would be a progress for the operation of the combustion process. Model-based control and optimization approaches by analysing the composition of the flue gas (Lange, 2007; Striūgas et al., 2017; Widder and Beckmann, 2017) or technologies for online fuel characterization such as near-infrared technology (Krämer et al., 2016) are reported in literature. Meiller et al. (2021) presented the development of a new sensor module for an enhanced fuel-flexible operation of biomass boilers. First approaches to gather information on the fuel and its volume in the feeding hopper in municipal solid waste incineration (MSWI) plants as well as the implementation of the gained data in combustion control system are published (Jell et al., 2021; Strobel et al., 2018). Warnecke et al. (2018) and Zwiellehner et al. (2018a, 2018b) could specify and validate a term for the calculation of the mass flow onto the grate in large-scale incineration plants. The obtained values were in a range up to ±10 wt% compared to the actual values of the plants. One important key parameter in the formula is the initial bulk density of the fuel in the feeding hopper. Furthermore, they identified a relationship between the bulk density and the CV for the ‘standard fuel mix’ of the MSWI plant ‘Gemeinschaftskraftwerk Schweinfurt’ (GKS). Based on theoretic considerations regarding the composition of the standard fuel mix, the relationship between bulk density and the CV was investigated for two variations. In the first, the ash content of the standard fuel mix was changed while the water content was set to a constant ratio at different CV. These conditions were reversed in the second variation. For both the cases, the resulting correlations between the bulk density and the CV differ marginally. Hence, Zwiellehner et al. (2018a) could specify an approximation formula for the bulk density of the standard fuel mix. In our research project ‘AdOnFuelControl’, the initial bulk density was calculated from the weight of the fuel captured by the crane weigher and the volume of the fuel was measured by a high-performance 3D laser scanner. In this paper, we characterize ‘fresh’ MSW, ‘aged’ MSW, RDF (fluff), RDF (fine grain), waste wood and dried, grained sewage sludge regarding use as fuel in large-scale incineration plants. This includes sampling, chemical analysis, testing the compression behaviour, initially testing with the 3D laser scanner and calculating the density in the feed hopper.

## Materials and methods

### Samples and large-scale sampling

**Figure 1.**
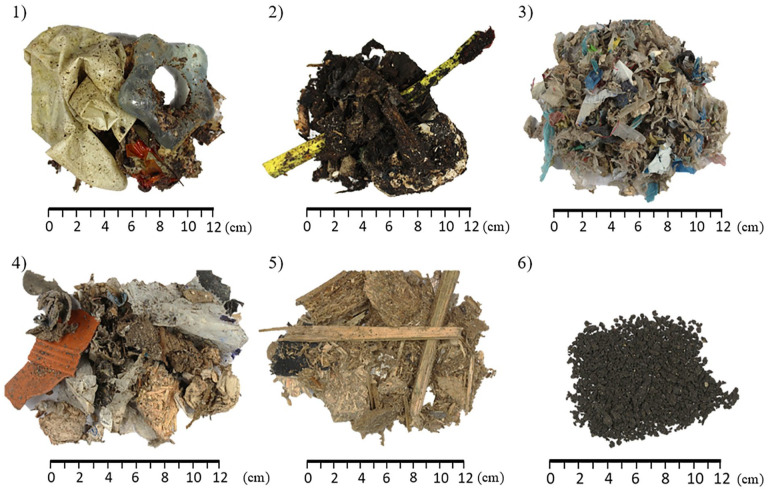
Investigated fuels: ‘fresh’ MSW (1), ‘aged’ MSW (2), RDF (fluff) (3), RDF (fine grain) (4), waste wood (5) and dried, grained sewage sludge (6).

The investigated fuels were provided by local suppliers or companies. Figure 1 gives an overview of the different samples. The samples ‘fresh’ MSW and ‘aged’ MSW were delivered in the bucket of a wheel loader (quantity used during experiments: 1.5–2.5 tonnes) from the on-site waste transfer station in Schweinfurt/Bergrheinfeld whereas the RDF (fluff and fine grain), waste wood and dried, grained sewage sludge came in a flexible intermediate bulk container (FIBC) with a volume of 1–2 m^3^. The fresh MSW came directly from the household (storage time at the waste transfer station around 2–3 days), while aged MSW was stored outdoors for around 2–4 months at the waste transfer station.

**Figure 2.**
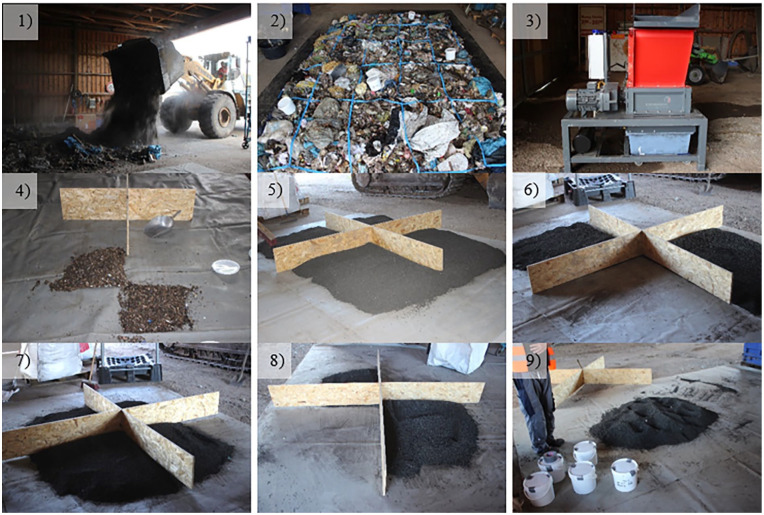
Processing of MSW for sampling (1–4) as well as sampling and narrowing of the dried, grained sewage sludge (5–9).

Depending on the form of delivery (bucket of the wheel loader or FIBC), two different approaches for sampling were realized. In the case of the MSW in the bucket, we followed the approach proposed by Danz et al. (2008). The MSW in the bucket of the wheel loader was discharged on a polyvinyl chloride (PVC)-coated canvas (compare Figure 2, (1), (2)) in the form of a fuel bed (length: 6 m, width: 4 m, height: approx. 0.3 m) and was divided into 25 even segments (five rows and five columns). In total, this procedure was executed two times for the fresh and the aged MSW. At each fuel bed, five segments were aggregated. One from each row and one from each column. Detailed information on the procedure can be found in Danz et al. (2008). Subsequently, the five segments were shredded to <20 mm in a one-shaft shredder (Model M600/1-600-5.5 kW; Co. Erdwich, 86859 Igling, Germany) (3). Inert and non-shreddable materials were separated and weighed. For all shredded materials of one fuel bed, four incremental samples with a volume of at least 1 L were taken. Four incremental samples were added to a collective sample and narrowed to a laboratory sample of at least 2 L (4). Buckets of 5.6 L were used and were almost filled. Accordingly, two laboratory samples were obtained from all shredded materials for fresh or aged MSW. The selected volumes by Danz et al. (2008) followed the specifications given in LAGA PN 98 (Länderarbeitsgemeinschaft Abfall, 2001). In addition, five segments were used for compression tests in the gantry press for each fuel bed, and the rest (2 × 15 segments per type of waste) were aggregated for a large-scale sieving experiment in a drum sieve. Following the compression tests, the MSW was divided into two parts. Each part was divided with a sample cross. The opposite quarters were shredded to <20 mm and sampled as just described. Hence, two laboratory samples were obtained for each fuel bed. In total, four laboratory samples were obtained for fresh and aged MSW. For the other types of fuel delivered in the FIBCs, a different proceeding was chosen. The contents of the FIBCs were emptied onto the PVC-coated canvas, mixed, coned and flattened to regular fuel beds (compare Figure 2, (5)). Then the fuels were narrowed with the help of a sample divider in the form of a cross. Two opposite quarters were used for the compressions tests (6). As just described, the other two opposite quarters were mixed and divided into four quarters again (7). Two opposite quarters were added in the gantry press (8). The other two opposite quarters were mixed again, and samples of at least 2 L were taken (9). On these samples, sieving experiments with a lab-scale drum sieve and chemical analysis were performed. The rest of the material was added to the gantry press. If necessary, a second FIBC was emptied and processed as mentioned earlier. The samples were analysed at an external laboratory for their fuel characteristics. The moisture content was analysed according to DIN 51718, the ash content at 900 °C was according to DIN 51719, the volatile compounds according to DIN 51720, the fixed carbon according to DIN 51734, the lower heating value (LHV) according to DIN 51900-1, the carbon, hydrogen and nitrogen content according to DIN 51732, the fluorine, chlorine and sulphur content according to DIN EN 14582 and the oxygen content according to DIN 51733.

### Volume measurements with a 3D high-performance laser scanner

**Figure 3.**
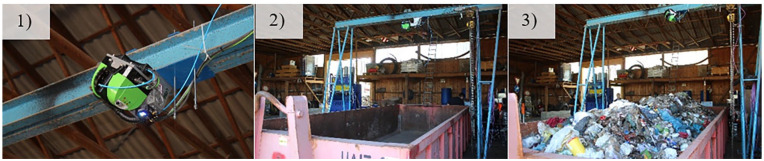
Images of the 3D high-performance laser scanner (1), the empty (2) and the filled (3) waste container.

In our experiments, volume measurements were conducted with the 3D high-performance laser scanner, LASE 3000D-C1-118 from Co. LASE, 46485 Wesel, Germany (compare Figure 3). The laser has a measurement range of 0.7 to >26 m at 10% target reflectivity, 1 to >80 m at 90% target reflectivity with a resolution of ±12 mm and a beam divergence of 4.7 mrad (LASE Industrielle Lasertechnik GmbH, 2014). The laser was connected to an industrial personal computer. For the initial tests, the laser scanner was attached to a small, portable gantry crane at a height of approximately 3 m and placed in the middle above the container. Subsequently, the volume of the MSW was measured in a waste container with a capacity of about 15 m^3^. First, the empty container was scanned and set to 0 m^3^. The volume was calculated from the difference between the empty and filled container. In addition, different disruptive factors such as the formation of shades, the influence of reflecting surfaces or dust as well as dirt on surfaces were investigated and the performance of the laser was tested.

### Compression tests of the samples and mathematical approach for the compression of bulk materials in a feed hopper

The compression test of the samples was performed in the gantry press PP 1207 (Co. Strautmann Umwelttechnik, 49219 Glandorf, Germany). During the experiments, a maximum pressure of about 144 bar in the hydraulic line is reached, which equals to a compression pressure of about 4.3 bar in the gantry press. The gantry press has a filling volume of about 0.84 m^3^. The pressure in the hydraulic line was logged on an LR8400-20 (Co. Hioki, 81 Koizumi, Ueda, Nagano 386-1192, Japan) with the help of the pressure transducer CK4500 (Co. Labom Mess- und Regelungstechnik, 27798 Hude, Germany) and the changing volume was recorded with the displacement sensor BTL-5-E10-M0100-B-32 (Co. Balluff, 73765 Neuhausen a.d.F., Germany).

As the basis for the compression of bulk materials in a feed hopper, we used the equation from Janssen (1895), which describes the forces in a silo. In Stieß (2009), a solution for the differential equation of the vertical pressure is presented as (equation (1)):

(1) $$p_{\mathrm{vf}}\left(z\right)=\frac{\rho_{\mathrm{Sch}}\cdot g}{K\cdot \mu }\cdot \frac{A}{U}\cdot \left(1-\exp\left(-K\cdot \mu \cdot \frac{U}{A}\cdot z\right)\right)$$

where *p*_vf_(*z*) is the vertical pressure, *z* is the distance measured from the surface of the bulk material, *ρ*_Sch_ is the bulk density, *g* is the gravity, *K* is the horizontal load ratio (0 for free-standing solid bodies and 1 for liquids, independent of *z*), *µ* is the wall friction coefficient (independent of *z*), *U* is the perimeter and *A* is the area. As *ρ*_Sch_ is dependent of *p*_vf_(*z*) and *A* and *U* are related to *z*, we substituted the term of equation (1) into the following equation (2):

(2) $$p_{\mathrm{vf}}(z)=\frac{\rho_{\mathrm{Sch}}(p_{\mathrm{vf}}(z))\cdot g}{K\cdot \mu }\cdot \frac{A\left(z\right)}{U\left(z\right)}\cdot \left(1-\exp\left(-K\cdot \mu \cdot \frac{U\left(z\right)}{A\left(z\right)}\cdot z\right)\right)$$

For solving equation (2), we selected an iterative approach. For starting the calculation of *ρ*_Sch_ (*z* = 0), the initial bulk density from the compression tests was used, and *p*_vf_ (*z* = 0) was set to 0. In the next step, *p*_vf_(*z_n_*)_*i*_ is calculated according to equation (2), by using the initial bulk density in the first iterative step *i*. The new *ρ*_Sch_(*z_n_*)_*i*+1_ is calculated according to equation (3) with *ρ*_Sch,initial_ = 275 kg/m^3^; subsequently, *p*_vf_(*z_n_*)_*i*+1_ is achieved:

(3) $$\begin{array}{c} \rho_{\mathrm{Sch}}\left(p_{\mathrm{vf}}\left(z\right)\right)=503,\mathrm{784}\cdot {\left(p_{\mathrm{vf}}\left(z\right)\right)}^{5}-\mathrm{574},030\cdot {\left(p_{\mathrm{vf}}\left(z\right)\right)}^{4}\\ +\;\mathrm{244},\mathrm{12}0\cdot {\left(p_{\mathrm{vf}}\left(z\right)\right)}^{3}+\mathrm{48},\mathrm{263}\cdot {\left(p_{\mathrm{vf}}\left(z\right)\right)}^{2}\\ +\mathrm{4996}.6\cdot p_{\mathrm{vf}}\left(z\right)+\rho_{\mathrm{Sch},\mathrm{inital}} \end{array}$$

After the changes from *p*_vf_(*z_n_*)_*i*,final−1_ to *p*_vf_(*z_n_*)_*i*,final_ are smaller than selected criteria, in our case <0.0001, the next *p*_vf_(*z_n_*_ + 1_) is determined with the help of the iterative value *ρ*_Sch_(*z_n_*)_*i*,final_. For *A*(*z*) and *U*(*z*), *z*-dependent terms were set up, taking into account the dimensions of the feed hopper of the MSWI plant GKS. For the first assessment of the pressure changes, the slope of the feed hopper was neglected. In our calculations, we selected a value of 0.56 for the horizontal load ratio *K* and of 0.47 for the wall friction coefficient.

## Results and discussion

### Chemical analysis

**Figure 4.**
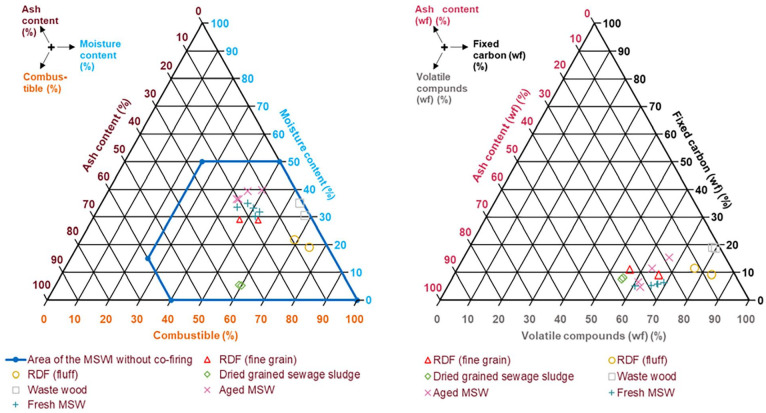
Fuel characteristics of the investigated samples in the Tanner diagram (left) and in a ternary diagram (right).

After sampling, the different fuels were analysed chemically. Figure 4 depicts the fuel characteristics of the investigated samples in the Tanner (left) and in a ternary diagram (right).

RDF (fine grain), old MSW and fresh MSW lie in a similar area, differing mainly in the moisture content (compare Figure 4, left). Dried, grained sewage sludge, RDF (fluff) and waste wood show higher contents of combustible (about 59–75%) compared to the other three (about 43–53%). Despite the higher content of combustibles, the three fuels differ considerably with regard to the percentage of volatile compounds and fixed carbon (compare Figure 4, right). Although the percentage of volatile compounds for RDF (fluff) and waste wood is around 80%, dried-grained sewage sludge provides around 55% of volatile compounds. Hence, the combustion behaviour will differ noticeably, as expected. Another key factor influencing the combustion behaviour is the different moisture content. In case of RDF (fine grain), old MSW and fresh MSW, the combustion behaviour is likely to be predominantly influenced by the different moisture contents, as most of the value for the volatile compounds range from 60 to 70% with fixed carbon values between 5 and 15%.

**Figure 5.**
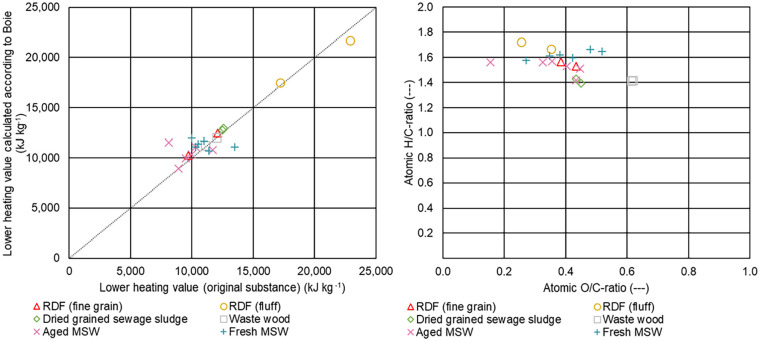
Comparison of the lower heating values (left) and van Krevelen diagram of the investigated samples (right).

Figure 5, left, shows a comparison of the LHVs obtained from the chemical analysis of the fuels and the heating value calculated according to the formula of Boie (compare equation (4)) (Danz et al., 2008):

(4) $$\begin{array}{c} \mathrm{LHV}=35\cdot \xi_{C}+94.3\cdot \xi_{H}+10.4\cdot \xi_{S}+6.3\cdot \xi_{N}\\ -\left(10.8\cdot \xi_{O}+3.4\cdot \xi_{\mathrm{Cl}}+6.4\cdot \xi_{F}+2.44\cdot \xi_{H_{2}O}\right) \end{array}$$

where *ξ* is the percentage of the particular element (C: carbon, H: hydrogen, S: sulphur, N: nitrogen, O: oxygen, Cl: chlorine, F: fluorine and H_2_O: moisture content).

For the calculation of the heating values according to the formula of Boie, the elemental composition received from the chemical analysis of the fuels was deployed. The dotted line in Figure 5, left, indicates the points where the analysed lower heating values equal the calculated ones. Most of the values lie near the dotted line indicating that for a first approach a calculation according to equation (4) is suitable. No obvious tendency of over- or underestimation is observed. If only the elementary content of the fuel is known from analysis and the calorific value has not been investigated, the formula, according to Boie, offers a good possibility to approximate the calorific value.

The right part of Figure 5 shows a van Krevelen diagram of the investigated samples, where the atomic H/C-ratio is plotted over the atomic O/C-ratio. As expected, waste wood with a H/C-ratio of 1.4 and an O/C-ratio of 0.6 is in the typical area of biomass (Jenkins et al., 1998; Tarafdar and Sinha, 2019; van Krevelen, 1993). Dried-grained sewage sludge also is with an H/C-ratio of around 1.4 and an O/C-ratio of 0.4. For the rest of the samples (fresh MSW, old MSW, RDF (fine grain) and RDF (fluff)), most of the values for H/C-ratios lie approximately in a range from 1.5 to 1.7 and for the O/C-ratios between 0.3 and 0.5. Lin et al. (2017) determined H/C-ratio of about 1.6 for polyethylene and polypropylene and O/C-ratio of about 0.05.

**Figure 6.**
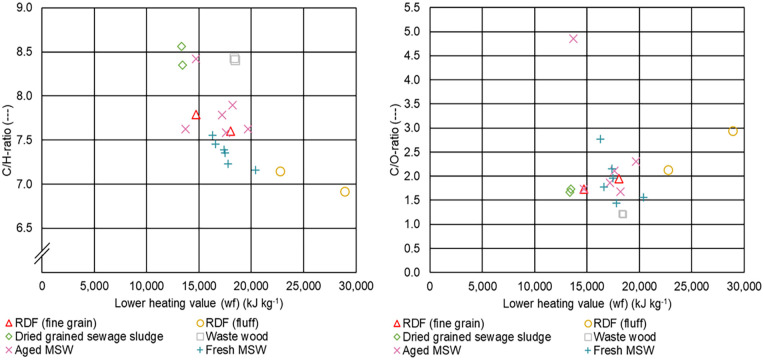
Relationship between C/H-ratio (left) and C/O-ratio (right) and the lower heating value for the different samples.

Figure 6 presents the relationship between C/H-ratio (left) and C/O-ratio (right) and the LHV for the different samples.

Most of the C/H-ratios for RDF (fine grain), RDF (fluff), aged MSW and fresh MSW lie in a range between 7 and 8, a typical range for this type of fuel (Beckmann et al., 2009; Rotter et al., 2011). For waste wood and dried-grained sewage sludge, the values lie around 8.5. In case of RDF (fine grain), aged MSW and fresh MSW, most of the C/O-ratios for LHVs waterfree (wf) between 15,000 and 20,000 kJ kg^−1^ lie between 1.5 and 2.0, which is in good accordance with the results presented in Beckmann et al. (2009). Also, in accordance with Beckmann et al., we observed that higher LHVs, as is in the case for RDF (fluff), tend to result in higher C/O-ratios, but the significance is limited as there are just two samples.

### Volume measurements with a 3D high-performance laser scanner

**Figure 7.**
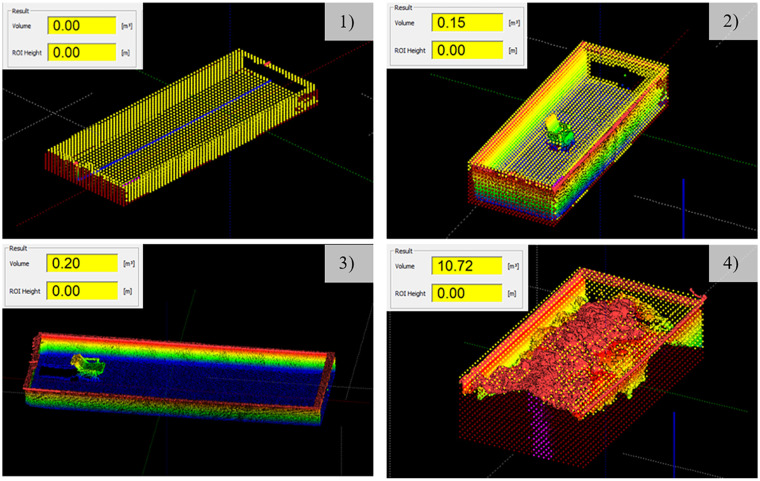
Images and 3D images of the empty (1), the partially filled (2, 3) and the filled (4) waste container.

Reflecting materials such as aluminium foil did not interfere with the laser. Small objects such as yardsticks can be detected by the laser. Dust (especially, finer powder) can interfere with the laser measurement. The laser is equipped with a pollution detection system, and therefore possible attachments on the lenses are recorded and can easily be identified. In addition, the lenses can be cleaned automatically by compressed air. Hence, long service life is expected despite rough conditions in the waste bunker in the MSWI plant. Figure 7 presents images and 3D images of the empty (1), the partially filled (2, 3) and the filled (4) waste container. In the trials, the formation of shades is shown to be the main disruptive factor. Depending on the position of the same object, different volumes are measured (2, 3). In our case, the volume of a chair was between 0.15 and 0.2 m^3^ depending on the position. When the container was filled with aged waste, the volume was 10.7 m^3^.

### Compression tests for samples and compression of bulk materials in a feed hopper

**Figure 8.**
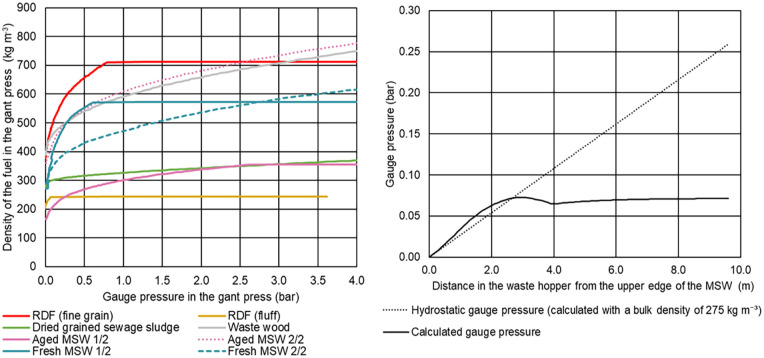
Compression behaviour of the investigated samples (left) and results of a calculation of the pressure changes in the feed hopper (right) of the MSWI plant GKS.

Figure 8 depicts the compression behaviour of the investigated samples (left) and provides the results of a calculation of the pressure changes in the feed hopper (right) of the MSWI plant GKS. In case of the compression tests, the bulk densities are plotted over the pressures in the gantry press. When curves are parallel to the axis of abscissae, the mechanical stop is reached. Hence, no further compression of the investigated sample is possible. In case of fresh MSW, a similar initial bulk density was observed, whereas for aged MSW, a higher discrepancy between the two samples was identified. One sample of the aged MSW is in the range of RDF (fine grain), and one sample is in the range of RDF (fluff) with regard to the compression behaviour, indicating that perhaps these types or similar were present in the aged MSW as fractions.

The right part of Figure 8 shows the results of a calculation of the pressure changes in the feed hopper. In addition, the hydrostatic gauge pressure calculated with a bulk density of 275 kg m^−3^ is plotted for a better comparison. Due to the varying dimensions of the feed hopper, the course of the curve also results in a decrease in pressure. Similar results for cones are reported in the literature (Schulze, 2019; Stieß, 2009). Zwiellehner et al. (2018a) specified a pressure of around 0.105 bar in the feeding area, which is slightly higher than our calculations. Whether the calculated values are valid for the real conditions in the feed hopper is not clear and need further research to validate.

### Characterization of different fuels for an advanced online fuel control system designed for large-scale incineration plants

**Figure 9.**
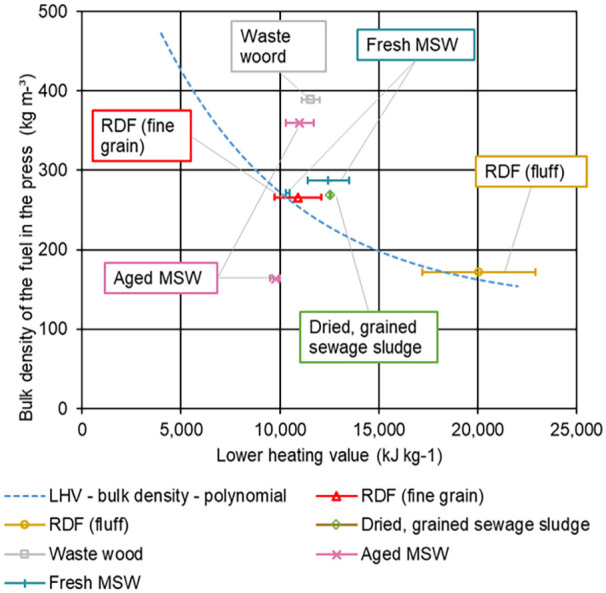
Classification of the investigated samples in the LHV – bulk density – polynomial from Zwiellehner et al. (2018a).

Figure 9 provides a classification of the investigated samples in the LHV – bulk density – polynomial from Zwiellehner et al. (2018a). The values for the initial bulk densities of the RDF (fine grain) and RDF (fluff) differ from the ones presented in the previous section, as the compressions tests were not performed instantly. The values obtained for RDF (fine grain), RDF (fluff), fresh MSW and dried, grained sewage sludge tend to confirm a correlation between bulk density and LHV. Especially if uncertainties would be added to the LHV – bulk density – polynomial and hence forming a band rather than a line. For the investigated samples of waste wood and aged MSW, the correlation seems to be valid to a very limited extent.

### Further actions

For large-scale experiments, the laser will be used above the feed hopper. Together with the weight of the MSW, the initial bulk density can be determined. Based on this, a specific energy content will be added to each waste layer. After an estimation of the compression, the amount of fuel entering onto the grate will be calculated hence offering an estimation of the input of energy into the firing unit. To validate this correlation, suitable parameters for the actual energy input into the firing have to be identified. Whether special temperature in the combustion chamber or the amount of fresh steam is more adequate will be part of the investigations during the large-scale experiments in the MSWI plant. In addition, changes in the combustion bed regarding the height and volume should be recognized and quantified during the combustion process to get information regarding the amount of volatile compounds and fixed carbon and the moisture content of the fuel. By providing all this information online in real-time, most likely, new opportunities will open up to regulate and control the combustion process. Whether the firing control can operate dynamically or different fixed control ranges are defined will be investigated in large-scale experiments.

## Conclusion

During our experiments, six different fuels were analysed chemically and their combustion behaviour including compression tests of the fuels was characterized. A 3D high-performance laser scanner was successfully used to quantify the volume of MSW. By measurements of the volume and the mass of the MSW in the large-scale plant GKS, the bulk density was calculated, which was linked to the LHV as presented by Zwiellehner et al. (2018a). In the next step, this correlation will be used to estimate the energy input into the firing under consideration of the density changes during the transport onto the grate. For the large-scale experiments in the GKS, adequate indicators have to be found to evaluate and identify varying LHV of the fuels and to quantify them. Also, changes in the combustion bed itself might reveal additional data for the firing control. In any case, the basic feasibility of the chosen approach could be shown during our experiments. The implementation of the 3D laser scanner and obtained knowledge as well as correlations will be the next decisive cruxes to succeed in an improved operation and regulation of the MSWI plant.
